# Assessing the metabolism of the olfactory circuit by use of ^18^F-FDG PET-CT imaging in patients suspected of suffering from Alzheimer’s disease or frontotemporal dementia

**DOI:** 10.1186/s13195-024-01604-7

**Published:** 2024-10-29

**Authors:** Daniël S. L. Loewenstein, Max van Grinsven, Cécile de Pont, Paul L. J. Dautzenberg, Astrid M. van Strien, Dylan Henssen

**Affiliations:** 1 Department of Medical Imaging, Radboud University Medical Center, Geert Grooteplein Zuid 10, Nijmegen, 6525 EZ The Netherlands; 2 Department of Medical Imaging, Jeroen Bosch Hospital, ‘s Hertogenbosch, The Netherlands; 3 Department of Geriatrics, Jeroen Bosch Hospital, ‘s Hertogenbosch, The Netherlands

**Keywords:** Alzheimer’s disease, Frontotemporal dementia, FDG PET, Neurodegenerative disorders, Neuroimaging, Olfactory system

## Abstract

**Purpose:**

The loss of olfactory function is known to occur in patients suffering from (behavioral variant) frontotemporal dementia ((bv)FTD) and Alzheimer’s disease (AD), although different pathophysiological mechanisms underpin this clinical symptom in both disorders. This study assessed whether brain metabolism of the olfactory circuit as assessed by positron emission tomography (PET) imaging with 2-[fluorine-18]fluoro-2-deoxy-d-glucose ([^18^F]-FDG) can distinguish these entities in different subsets of patients.

**Methods:**

Patients presenting with cognitive decline were included from a prospectively kept database: (1) bvFTD patients, (2) AD patients and (3) patients with logopenic primary progressive aphasia (PPA). Metabolic rates were calculated for different regions of the olfactory circuit for each subgroup and compared with a cohort of subjects with normal brain metabolism. Additionally, in patients with a logopenic PPA pattern on PET-imaging, statistical parametric mapping (SPM) analysis was performed.

**Results:**

The metabolism of subdivisions of the olfactory circuit as assessed by [^18^F]-FDG PET brain imaging to bvFTD and AD from control subjects resulted in sensitivity/specificity rates of 95/87.5% and 80/83.3%, respectively. A sensitivity/specificity rate of 100/87.5% was achieved when used to differentiate AD from bvFTD. In patients with the PPA pattern on imaging, the underlying cause (either FTD or AD) could be determined with a sensitivity/specificity rate of 88/82%. SPM analysis concurred that different regions of the olfactory circuit were affected in patients suffering from AD PPA or bvFTD PPA.

**Conclusion:**

Metabolic dysfunction in the olfactory circuit is different in various neurodegenerative disorders. Further investigation of the correlations between the cerebral metabolism and the mechanisms which drive olfactory dysfunction is needed.

## Introduction

Primary neurodegenerative disorders, characterized by accumulative damage to the brain, are the leading cause of dementia and are characterized by progressive, accumulative damage to the brain. Clinically, the deterioration of neuronal structures and interconnective circuits result in symptoms of memory deficits and impairment of higher cognitive functions, leading to social and functional dysfunction [1]. Alzheimer’s disease (AD) is the most common (50–60%) cause of dementia in older people and is one of the leading contributors to mortality in individuals over 65 years of age [1, 2]. It is classically known for its presentation with memory problems, but has subtypes such as the logopenic primary progressive aphasia (PPA) that mainly involves word-finding deficits. In younger individuals, frontotemporal dementia (FTD) is frequently encountered as a cause of dementia (15–25%). About half of the patients present with behavioral changes (behavioral variant FTD; bvFTD). The other half present with language deficits (PPA), typically characterized by either impaired speech production (progressive non fluent aphasia) or impaired word recognition and semantic memory (semantic dementia) [3, 4]. The logopenic PPA is typically associated with AD, however, other forms of PPA have been shown to be more often caused by FTD [5]. Next to the decline of cortical functions in dementia, olfactory dysfunction can also be observed clinically in AD, FTD and other neurodegenerative disorders [6]. In AD, olfactory function can be altered in ~ 85–90% of AD patients [7, 8]. According to previous reports, olfactory dysfunction occurs in up to ~ 96% of patients diagnosed with FTD [9–12]. The symptoms of olfactory dysfunction differ between AD and FTD, showing a more complete anosmia in FTD patients and an affected odor memory and odor identification in AD patients [6, 10, 13].

Early diagnosis and characterization of different neurodegenerative disorders is a growing challenge in medicine, as clinical symptoms show considerable overlap. The diagnosis of a primary neurodegenerative disorder can be made by investigating biomarkers in cerebrospinal fluid, Amyloid or Tau-PET and/or by excluding other causes (e.g., structural, vascular, toxic etiologies) by use of anatomic imaging (computed tomography (CT) and magnetic resonance imaging (MRI)) [14, 15]. Anatomic imaging modalities are, however, limited in their ability to help distinguish various forms of neurodegenerative disorders early on in the disease as atrophy and ventricular enlargement are late signs of neurodegeneration [16, 17]. Positron emission tomography (PET) imaging with [fluorine-18]fluoro-2-deoxy-d-glucose ([^18^F]-FDG) is known as a highly useful imaging modality to distinguish different neurodegenerative disorders, as each disorder is characterized by a metabolic signature pattern (for an overview, see [18]). Summarizing, early AD is characterized by a hypometabolism in the posterior cingulate gyrus [19] which advances to involve the precuneus and posterior parietal and temporal lobes. In more advanced cases, hypometabolism extends to involve the frontal lobe as well. Asymmetrical hypometabolism can be observed and it must be noted that different subtypes of AD show different patterns of hypometabolism [18]. In AD, a preserved metabolism is typical in the basal ganglia, thalamus, infratentorial brain structures and the anterior cingulate and visual cortices [18, 20–22]. In bvFTD, hypometabolism is observed in the frontal and anterior temporal lobes as well as in the anterior cingulate gyrus [23–25]. However, pattern recognition can be more elusive in patients suffering from progressive primary aphasia (PPA). PPA is a neurodegenerative clinical syndrome which is characterized by a prominent progressive language impairment which can be accompanied by other cognitive or neurological changes [26]. Histopathological studies showed evidence that cases of PPA frequently have an FTD or AD neuropathological substrate [27, 28]. This group of patients can be a challenge to diagnose by use of [^18^F]-FDG PET neuroimaging alone, specifically when the hypometabolic regions of the PPA is not accompanied by other signature hypometabolic regions that clearly differentiate AD from FTD. For this study, this occurred solely when the pattern was of a logopenic PPA (parietotemporal involvement), and could fit either an AD diagnosis or an FTD diagnosis.

Hypometabolism in specific regions of the brain is highly associated with the clinical dysfunction that is presented by the patient. Likewise, with clinical olfactory symptoms, we could expect to detect hypometabolism in related areas of the brain. Whether FDG PET can be used to quantitatively assess the olfactory deficits in patients suffering from AD/FTD, however, remains unknown. Thereby, it also remains elusive whether an abnormal metabolism of the olfactory brain can be used in the diagnosis of AD and/or bvFTD.

Therefore, the metabolism within the olfactory brain was retrospectively quantified in the prospectively collected database comprising AD-patients, bvFTD-patients and subjects with normal brain metabolic signatures. The developed statistical method was tested in a group of PPA-patients caused by either AD or FTD as an external validation.

## Materials and methods

### Ethical approval

The regional ethical review board (METC Brabant) waived ethical approval due to the retrospective nature of this study. Patients who did not provide consent to have their imaging data used in scientific research were not included.

### Patient selection

Patients were retrospectively included from a prospectively kept clinical database from Jeroen Bosch Hospital, Den Bosch, The Netherlands. Patients underwent [^18^F]-FDG PET brain imaging between 2011 and 2020. Three groups of patients were eligible for inclusion: (1) subjects with normal brain metabolism; (2) patients with metabolic patterns and clinical characteristics of bvFTD; and (3) patients with metabolic patterns and clinical characteristics of AD. Exclusion criteria were: patients younger than 18 years, patients who refused to share anonymized retrospective data for research purposes and patients who showed metabolic changes suggestive of other neurodegenerative disorders (e.g., Lewy Body Dementia, Corticobasal Degeneration). Additionally, a fourth group of 15 cases was included. In these patients, brain PET imaging revealed a logopenic PPA pattern and could not completely resolve the discussion of whether the patient suffered from bvFTD or AD. The final diagnosis was determined based on the clinical progression of the disease in the years following the acquisition of the [18F]FDG-PET imaging used in this study. Effort was devoted to forming a balanced cohort of patients exhibiting a logopenic PPA pattern, diverging from the typical epidemiological distribution of PPA variants [29]. Final diagnosis for all patients was based on a combination of clinical parameters, biomarkers in cerebrospinal fluid (when available, including tau, p-tau, and αβ amyloid), neuroimaging findings and disease progression over time. Clinical diagnosis was made by a board-certified neurologist or geriatrician based on the developed criteria for diagnosing frontotemporal dementia, as established by the International Behavioural Variant FTD Criteria Consortium, and Alzheimer’s dementia, as established by the National Institute on Aging and the Alzheimer’s Association [30, 31].

### Image acquisition

The imaging protocol at our center comprised a static imaging session, acquired in three-dimensional mode for improved sensitivity over a time period of 20 min, starting after 30 min of the intravenous injection of ~ 160 MBq [^18^F]-FDG. Patients were instructed to keep the head still during image acquisition, since movement of the head would interfere with image quality and compromise the validity of the attenuation correction algorithm. Serum glucose levels ranged between 4.1 and 9.2 mmol/L; 73.8-165.6 mg/dL (5.8 ± 0.9 mmol/L; 104.4 ± 16.2 mg/dL). All brain PET imaging was performed on a Siemens Biograph mCT scanner (Siemens Medical Solutions, Hoffman Estates, Ill). Low-dose CT was performed with the patient in the same bed position as for PET and the acquired data were used for attenuation correction as well as anatomic correlation.

### Post-processing and display

Following standard tomographic reconstruction with scatter correction and resolution recovery, CT-based attenuation correction was applied. Post-processing consisted of image reorientation in a canthocaudal orientation and normalization of uptake. Images were displayed in standard grey-scale in three anatomical planes (axial, coronal and sagittal). The software package syngo^®^.via (Neurology software package; www.healthcare.siemens.com) was used for quantification and standardization of metabolic processes in brain regions. By standardizing brain images, we accounted for individual variations in head size and shape and achieved correct alignments allowing for correlation with brain regions of available brain atlases. Within this study, PET data was mapped on a voxel-to-voxel basis to the Talairach standard brain template. Both two-dimensional cross-sectional images and three-dimensional surface-rendered images, all with voxel-based color coding, were created for all patients. The three-dimensional surface-rendered images are made by identification of the highest activity voxels along 13 mm predefined vectors which were angled perpendicular to the brain surfaces. The maximum activity was assigned to a surface voxel by use of back-projection. It is known that the combination of these reconstructions allows for enhanced pattern recognition [32].

### Regions of interest

FreeSurfer parcellation and segmentation of a standard MNI152-template brain was used to outline anatomically distinct regions of the cortex and dividing the sub-cortical nuclei into distinct structures. These parcellations were created along the lines of two atlases that come with FreeSurfer: The Destrieux atlas, and the Desikan-Killiany atlas. The parcellation and segmentation results were used to derive the regions of interest that constitute the olfactory brain and were subsequently translated to the spatially normalized patient data for statistical analysis.

**Fig. 1:**
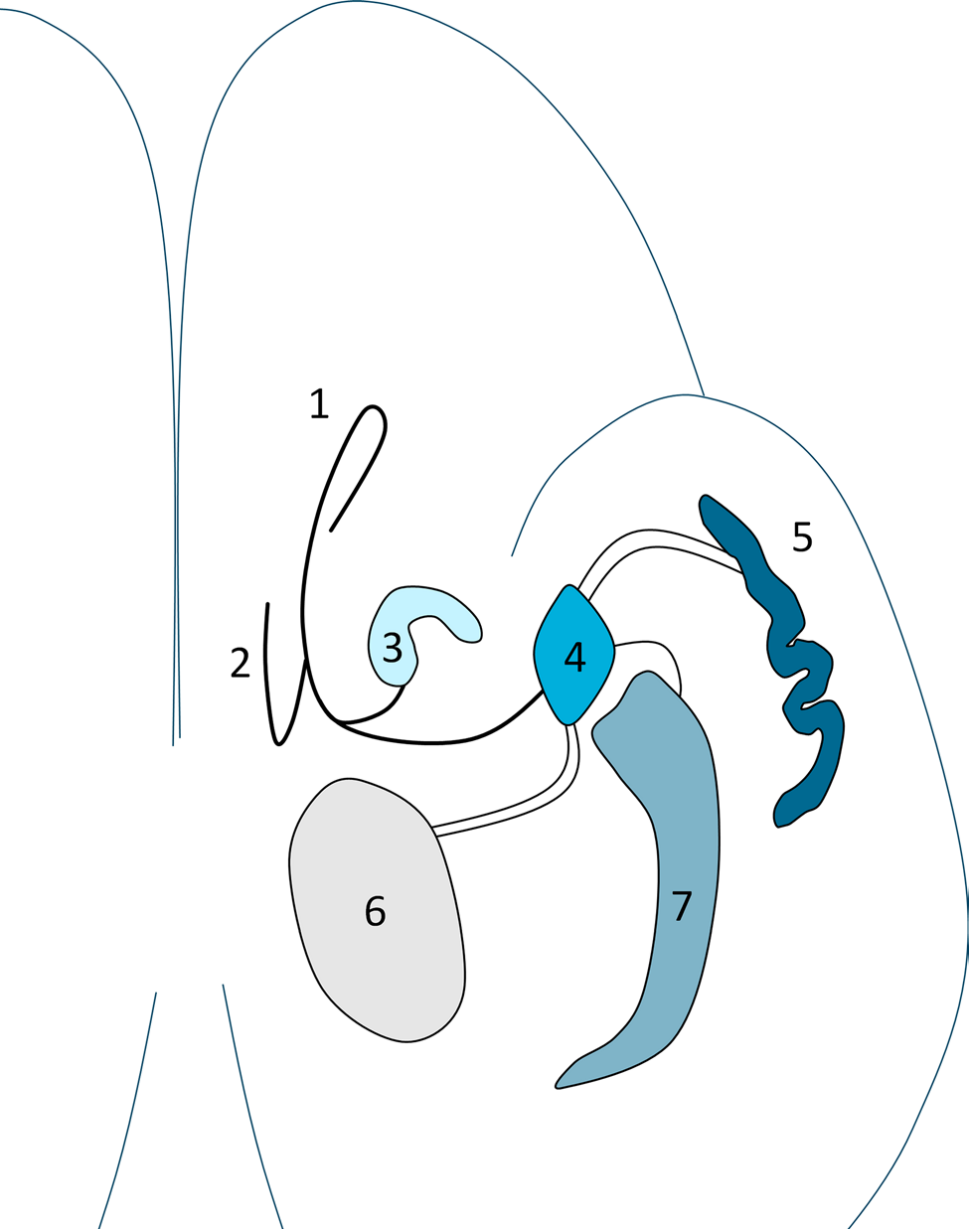
Schematic axial representation of cortical and subcortical structures which form the olfactory brain. 1 = Olfactory bulb; 2 = Lateral olfactory stria; 3 = Primary olfactory cortex; 4 = Amygdaloid complex; 5 = Insular cortex; 6 = Thalamus complex; 7 = Hippocampus

The olfactory brain comprises the olfactory bulb, the olfactory cortex (i.e., the entorhinal cortex), the amygdaloid complex and the insula. It is well established that efferent projections connect with primary structures of the limbic system, including the hippocampus and the thalamus [33, 34]. The olfactory bulbs are small flattened ovoid bodies on the basal aspect of the brain. At the site of attachment, the olfactory bulb bifurcates in medial olfactory stria and lateral olfactory stria. The medial olfactory stria extends towards the subcallosal area; the lateral olfactory stria courses towards the olfactory cortex and the insula. The olfactory cortex and insula interconnect with the amygdala, the hippocampus and thalamus [34]. A schematic overview is provided in Fig. 1. Two metrics were derived from the regions of interest: mean uptake (body weight SUVRs) and Z-scores. Regarding the mean uptake per region, [^18^F]FDG PET intensity normalization was carried out by using the pons as a reference region for each individual patient, following the methodology as described by Verger et al. [35]. By employing this frequently used protocol, we enhanced the comparability of our results with those of other studies. The Z-score represents distance from the estimated population mean relative to the estimated population standard deviation (e.g. the number of standard deviations from the population mean), which allows for differences in variation in the normal population in different regions of the brain. The estimated population mean and estimated population standard deviation are documented by Siemens in the Syngo database. The Syngo database comprises scans from confirmed normal individuals, thereby offering a validated sample for deriving the estimated population mean and standard deviation [36].

The here described ROI-based analysis was considered essential to observe differences in glucose metabolism in relatively small regions of the brain which are involved in olfactory input processing between subjects with normal brain metabolism and patients suffering from bvFTD or AD. Voxel-based analyses such as statistical parametric mapping was believed unable to elucidate these changes in brain metabolism as the large differences between the metabolism in different canonical brain regions involved in either AD or bvFTD (e.g., posterior cingulate cortex, medial frontal cortex) would easily overshadow relative small clusters of metabolic changes in the brain regions involved in olfactory function. For that reason, changes in glucose metabolism in olfactory-associated regions between subjects with normal brain metabolism and patients suffering from bvFTD or AD were assessed using an ROI-based approach. Nonetheless, to validate the findings of the ROI-based analysis, a group of PPA patients was analyzed using a rigorous, SPM-based analysis which is described in detail hereafter.

### Statistical analysis

Analysis was performed using IBM SPSS Statistics version 25 (*IBM Corp. Released 2017. IBM SPSS Statistics for Windows*,* Version 25.0. Armonk*,* NY: IBM Corp.*). Descriptive statistical analyses were represented as normalized mean uptake values per region of interest (SUVR-values). The SUVR-values were accompanied by the corresponding Z-scores per region of interest. Both integrals were assessed per group by use of the independent Student’s t-test. Statistical tests were two-sided and considered significant if *P* < 0.05. Bonferroni correction for multiple testing was carried out.

Receiver operator characteristics (ROC) curves were plotted to assess the diagnostic ability of a binary classifier system for the Z-score maps of each region of interest. Diagnostic accuracy was provided for each region as area under the curve (AUC). Groups which were compared comprised (1) bvFTD vs. normal, (2) AD vs. normal and (3) bvFTD vs. AD. Optimal cut-off values for the different discriminating abilities were calculated. The optimal cut-off value derived from the analysis to distinguish bvFTD and AD was tested in the fourth group of patients (*n* = 15). In this subgroup, true positive (TP), false positive (FP), true negative (TN) and false negative (FN) rates were calculated, using clinical outcome over time as the silver standard diagnosis, given that the gold standard of histopathological analysis of brain tissue was unavailable for this patient cohort.

### Statistical parametric mapping

To perform statistical parametric mapping (SPM) analyses, PET-images of the PPA patient cohort were post-processed using the statistical parametric mapping (SPM) 12 program (Wellcome Department of Imaging Neuroscience Group, London, UK; http://www.fil.ion.ucl.ac.uk/spm) [37].

First, all images were re-oriented to be aligned with the anterior commissure. Inter-subject alignment was achieved by use of a discrete cosine transform model. To spatially normalize the subjects to MNI space, the normalization module available in SPM12 was used. Next to normalization of data to the MNI space, this algorithm warps the individual subjects to fit a standardised template. More specifically, we used the Dementia-Specific [^18^F]-FDG-PET template for SPM normalization [38]. Images were not modulated, in order to preserve diagnostic accuracy [39]. Data were smoothed with a standard 8-mm FWHM gaussian kernel to increase signal-to-noise ratio [40].

A standard relative threshold of 0.8 was applied, excluding any voxels with an intensity exceeding 80% of the mean global value of the participant. Overall, grand mean scaling was set at the default setting of 50 in order to create a more intuitive scale without influencing statistical analysis [41]. Proportional normalisation was achieved through a mean global calculation. Additionally, the brain masks within the FieldMap toolbox of SPM12 served as an explicit mask at this stage in the analysis [38]. To assess differences in voxel intensities between groups of FDG-PET data, a general linear model was created, using an independent two-sample t-test. Statistical outcomes acquired from statistical parametric mapping were uncorrected for multiple comparisons (statistical significance was set at *P* < 0.001) and the voxel threshold was extended to 20 voxels, disregarding any clusters smaller than 20 voxels in size. Final results were visualised with the xjView toolbox (https://www.alivelearn.net/xjview).

In the context of SPM analysis for PET images, the most commonly employed method for intensity normalization within the SPM pipeline is proportional scaling [42, 43]. Additionally, the specific pipeline utilized in this study has been shown to significantly improve the discrimination between AD and FTD in a clinical setting [44].

## Results

### Descriptive analysis

In total, 75 patients (34 females; 66.4 ± 7.9 years) were included. Sixteen patients suffered from bvFTD; 24 patients suffered from AD. Twenty patients were included with a normal cerebral metabolism. Additionally, 15 cases in which brain PET imaging revealed a logopenic PPA pattern and could not aid to determine the causative disease were included. Mean standardized uptake values (SUVRs) and Z-scores of the amygdala (L/R), hippocampus (L/R), insular cortex (L/R), olfactory cortex (L/R) and thalamus (L/R) for each subgroup are provided in Table 1.

**Table 1:** Overview of the included groups

Group	Sex (M/F)	Mean age (± SD) (years)	Amydala left		Amydala right		Hippocampus left		Hippocampus right		Insular cortex left		Insular cortex right		Olfactory cortex left		Olfactory cortex right		Thalamus left		Thalamus right	
			Mean SUV	Z-score	Mean SUV	Z-score	Mean SUV	Z-score	Mean SUV	Z-score	Mean SUV	Z-score	Mean SUV	Z-score	Mean SUV	Z-score	Mean SUV	Z-score	Mean SUV	Z-score	Mean SUV	Z-score
Normal metabolism (*n* = 20)	11/9	62.2 ± 9.4	1.40	0.4	1.35	0.6	1.40	0.1	1.34	0.3	1.84	-0.1	1.79	-0.2	1.45	-0.1	1.45	-0.2	1.97	-0.3	1.94	-0.3
bvFTD (*n* = 16)	12/4	67.6 ± 5.5	0.9	-1.7	0.84	-1.8	0.88	-2.2	0.89	-2.3	1.20	-2.6	1.18	-2.2	0.91	-2.3	0.89	-2.3	1.40	0.2	1.38	-0.9
AD (*n* = 24)	10/13	67.2 ± 8.5	1.17	1.4	1.15	1.7	1.13	-0.3	1.22	0.1	1.52	0.2	1.53	0.7	1.25	0.5	1.25	0.3	1.57	0.02	1.54	-0.3
PPA (*n* = 15)	8/7	70.5 ± 5.2	1.40	0.6	1.35	1.0	1.40	-1.2	1.33	-0.8	1.84	-1.4	1.79	-0.8	1.45	-0.4	1.45	-0.6	1.97	-1.5	1.94	-1.4

AD = Alzheimer’s disease; bvFTD = Behavioral variant frontotemporal dementia; PPA = Primary progressive aphasia; SUV = Standardized uptake value

### Comparison of bvFTD patients vs. normal subjects

When comparing the normalized mean SUVRs in the amygdala, the hippocampus, the thalamus, the insular cortices and the olfactory cortices, a significantly lower uptake of FDG was observed in these regions in bvFTD patients when compared to normal subjects (both left and right regions, all *P* < 0.05). When comparing the Z-score of the aforementioned regions, only the bilateral thalamus was found not significantly different between these subjects (left thalamus *P* = 0.254; right thalamus *P* = 0.431). All other regions (both left and right) showed significant differences in Z-scores (all *P* ≤ 0.001).

**Fig. 2:**
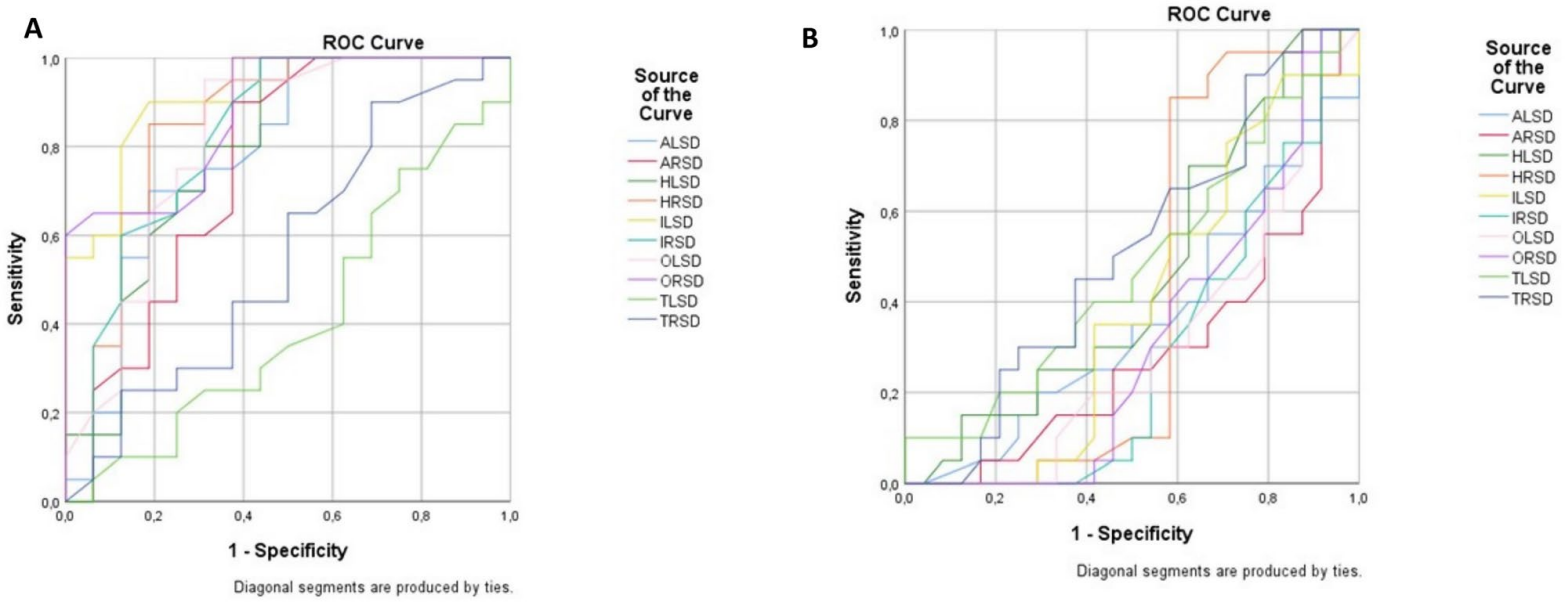
ROC-curves of the Z-score per studied region to distinguish bvFTD patients from patients with normal cerebral metabolism (**A**) and AD patients with normal cerebral metabolism (**B**). AD = Alzheimer’s disease; ALSD = Z-score of the left amygdala region; ARSD = Z-score of the right amygdala region; bvFTD = Behavioral variant frontotemporal dementia; HLSD = Z-score of the left hippocampus region; HRSD = Z-score of the right hippocampus region; OLSD = Z-score of the left olfactory cortex region; ORSD = Z-score of the right olfactory cortex region; ILSD = Z-score of the left insular cortex region; IRSD = Z-score of the right insular cortex region; ROC = Receiver-operator characteristics; SUVR = Normalized standardized uptake value; TLSD = Z-score of the left thalamus region; TRSD = Z-score of the right thalamus region

Regarding Z-scores, ROC curve analysis provided AUC values of 0.786/0.753, 0.794/0.845 0.919/0.822, 0.816/0.878 and 0.413/0.556 for the left/right amygdala, hippocampus, insular cortex, olfactory cortex and thalamus, respectively (Fig. 2A). When combining the Z-scores of the aforementioned regions, an optimal sensitivity/specificity rate of 95/87.5% with an AUC of 0.913 is achieved with regard to distinguishing FDT from normal subjects.

### Comparison of AD patients vs. normal subjects

When comparing normalized mean SUVRs in the aforementioned regions of interest, no significant difference in uptake of FDG was observed between AD patients and normal subjects. When comparing Z-score of the different regions, only the right insular cortex and the right- and left olfactory cortices showed significantly lower values (*P* = 0.015, *P* = 0.035 and *P* = 0.05, respectively) in AD patients when compared with normal subjects. All other regions showed no significant differences in Z-scores.

**Fig. 3:**
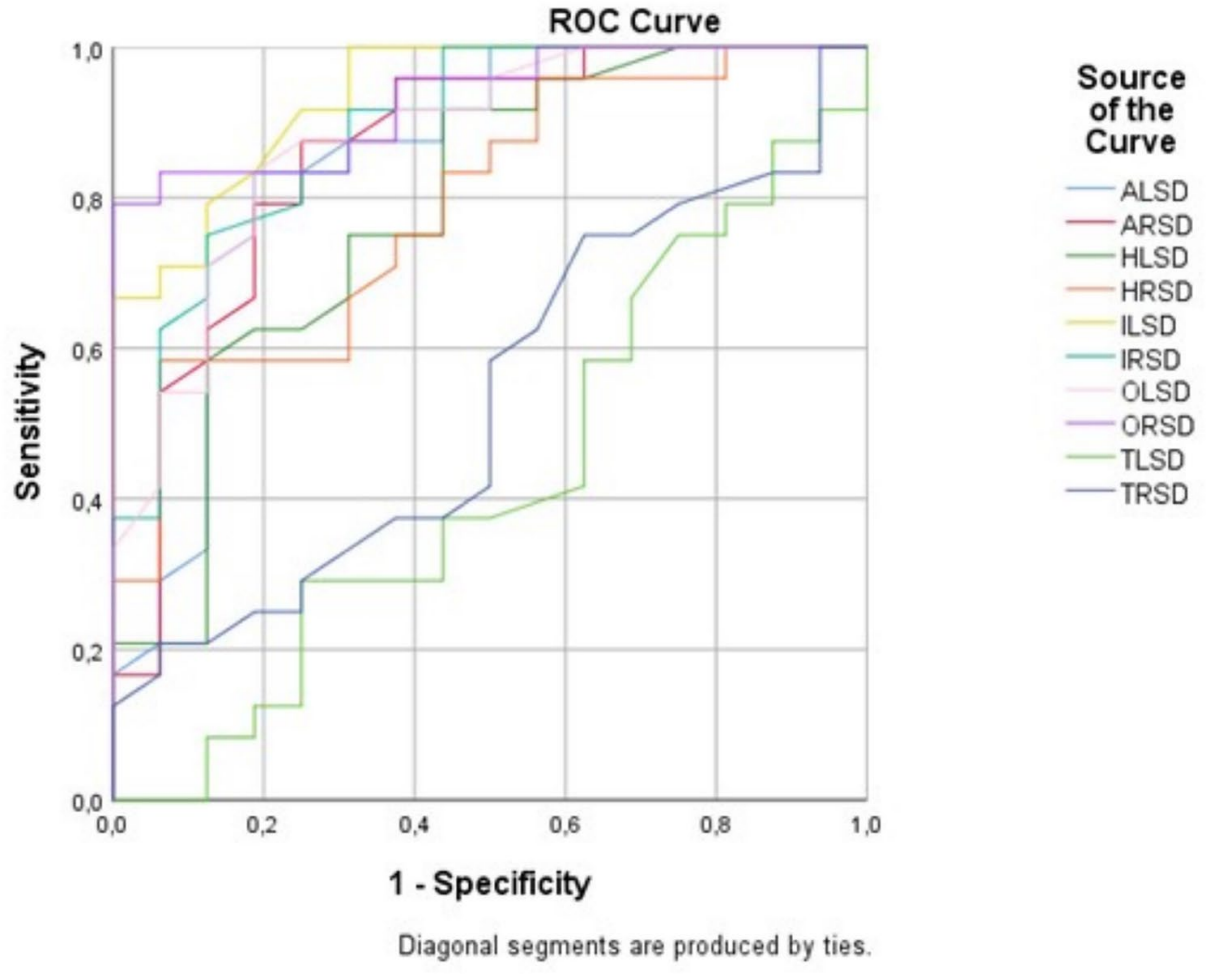
ROC-curve of Z-score per studied region to distinguish bvFTD patients from AD patients. AD = Alzheimer’s disease; ALSD = Z-score of the left amygdala region; ARSD = Z-score of the right amygdala region; bvFTD = Behavioral variant frontotemporal dementia; HLSD = Z-score of the left hippocampus region; HRSD = Z-score of the right hippocampus region; OLSD = Z-score of the left olfactory cortex region; ORSD = Z-score of the right olfactory cortex region; ILSD = Z-score of the left insular cortex region; IRSD = Z-score of the right insular cortex region; ROC = Receiver-operator characteristics; SUVR = Normalized standardized uptake value; TLSD = Z-score of the left thalamus region; TRSD = Z-score of the right thalamus region

When analyzing the accuracy of the Z-score of the different regions, AUC values were 0.358/0.290, 0.460/0.414, 0.390/0.290, 0.307/0.316 and 0.478/0.511 for the left/right amygdala, hippocampus, insular cortex, olfactory cortex and thalamus, respectively (Figs. 2B and 3B). Clearly, the individual ROIs perform poorly in predicting subjects with Alzheimer’s disease (AD), as indicated by AUC values well below 0.5. However, this is interpreted as suggesting a strong ability to identify healthy controls, which is reflected by the inverse of the results presented in this analysis. When combining the Z-scores of the aforementioned regions, an optimal sensitivity/specificity rate of 80/83.3% with an AUC of 0.817 is achieved with regard to distinguishing AD from normal subjects.

### Comparison of bvFTD patients vs. AD patients

Based on the normalized mean SUVR per region, the amygdala (left and right; *P* = 0.026 and *P* = 0.011, respectively), hippocampus (left and right; *P* = 0.040 and *P* = 0.013, respectively), right insular cortex (*P* = 0.047) and olfactory cortex (left and right; *P* = 0.022 and *P* = 0.015, respectively) showed significant differences between bvFTD and AD patients with significantly lower normalized values in bvFTD patients. Other regions showed no significant differences in mean SUVR between bvFTD and AD patients.

When using Z-scores, on the other hand, all regions with the exception of the bilateral thalamus showed significantly decreased uptake patterns in bvFTD patients (*P* ≤ 0.001). More specifically, bvFTD patients showed significantly lower Z-scores, indicating more seriously disturbed metabolism in the investigated regions. The Z-score of the left/right thalamus showed to be similar in bvFTD and AD patients (*P* = 0.268 and *P* = 0.578, respectively).

When analyzing the accuracy of the Z-score of the different regions, AUC values of 0.850/0.861, 0.784/0.788, 0.936/0.889, 0.878/0.930 and 0.430/0.534 for the left/right amygdala, hippocampus, insular cortex, olfactory cortex and thalamus, respectively (Fig. 3). When combining the Z-scores of the aforementioned regions, an optimal sensitivity/specificity rate of 100/87.5% with an AUC of 0.938 is achieved with regard to distinguishing AD from bvFTD subjects.

### Defining the FTD vs. AD origin of the logopenic PPA pattern

Regions in which Z-score showed significant results at very high levels of significance (*P* < 0.001) and with high AUC values (> 0.850) were included in this sub-analysis as diagnostic markers of differences in cerebral metabolism between indistinct cases of FTD and AD. These regions concerned the bilateral amygdala, the bilateral insular cortex and bilateral olfactory cortex. In all aforementioned regions, the Z-score was lower in FTD PPA patients as compared with the Z-score in AD PPA patients. Therefore, values higher than the reported cut-off values should be considered as a reflection of AD pathology.

ROC analysis of the left amygdala resulted in an optimal cut-off value of Z-score of -0.65 with a corresponding sensitivity/specificity rate of 76/64%. Optimal cut-off value of Z-score for the right amygdala was at a Z-score of 0.95 with corresponding sensitivity of 70% and specificity of 83%.

ROC analysis showed optimal cut-off values of Z-score of -1.35/-1.0 of the left and right insular cortex, respectively. These values resulted in a sensitivity/specificity of 82/73% and 85/73% for the left and right insular cortex, respectively. For the Z-score value of the left olfactory cortex, an optimal cut-off value of -0.6 resulting in a sensitivity/specificity of 76/73%. With a cut-off value of -0.4 of the right olfactory cortex, a sensitivity of 73% and specificity of 91% was achieved.

When combining the discriminative effects of the Z-score values of the left/right amygdala and the left/right olfactory and insular cortices, an AUC of 0.926 was achieved, with a sensitivity/specificity rate of 88/82%.

### SPM results

**Fig. 4:**
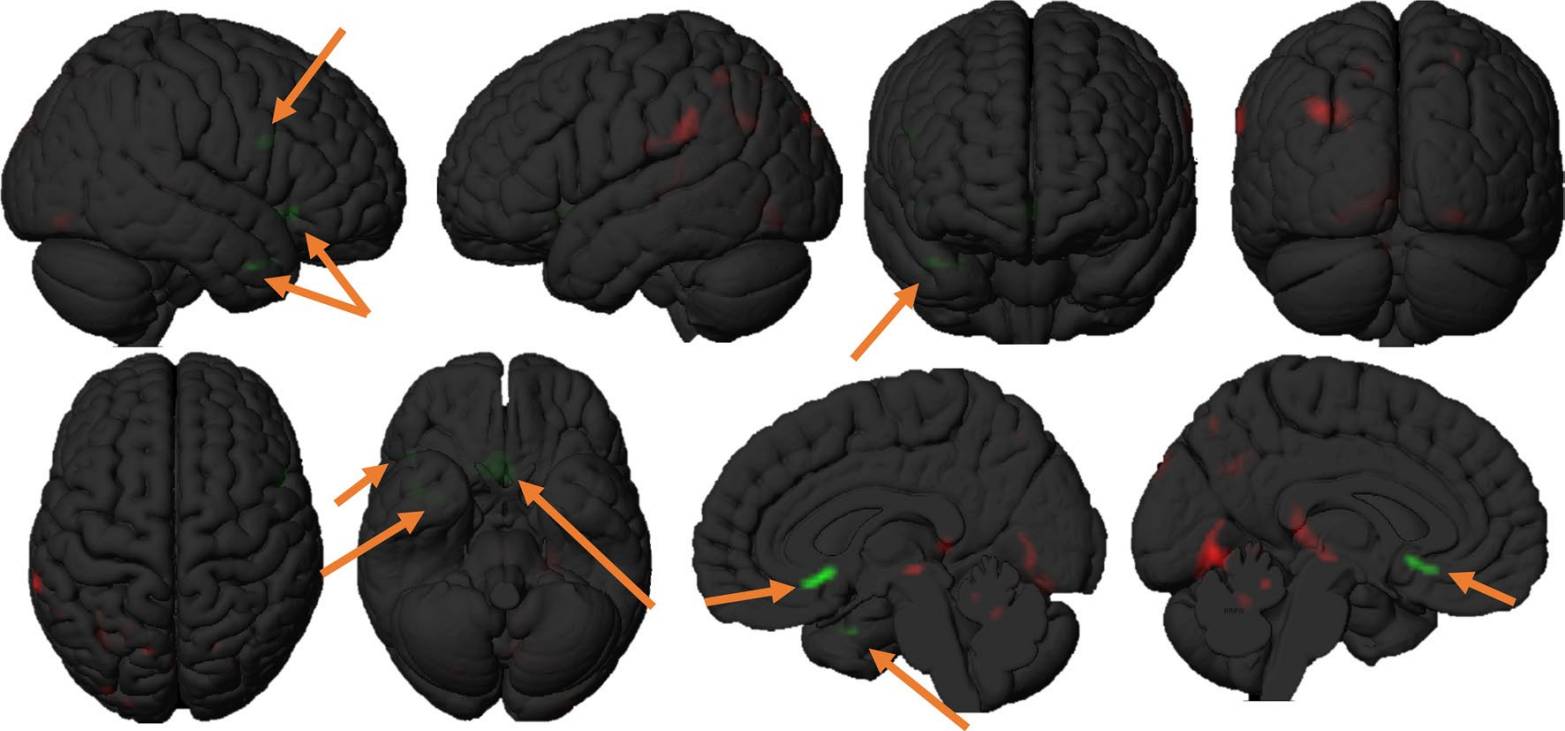
Regional [^18 ^F]-FDG PET hypometabolism in bvFTD PPA patients compared to AD PPA patients. Regions of significant differences in hypometabolism between bvFTD PPA patients and AD PPA patients were analysed using conjunction analysis. Results shown at *P* < 0.001. AD = Alzheimer’s disease; bvFTD = Behavioral variant frontotemporal dementia; PPA: primary progressive aphasia. It can be appreciated that in bvFTD PPA patients, a significantly lower metabolism was observed in the bilateral olfactory cortex, right temporal pole and right superior frontal gyrus (green clusters, indicated with gold arrows) compared to the metabolism observed in AD PPA patients. In AD PPA patients, on the other hand, a significantly lower metabolism was observed in the bilateral parahippocampal gyrus, bilateral lingual gyrus, left posterior cingulate cortex, left supramarginal gyrus, left superior parietal lobule. Additionally, a significantly lower metabolism was observed in AD PPA patients central in the midbrain, located in the region of the mamillotegmental tract

SPM analysis showed significant hypometabolism in the superior frontal gyrus, middle frontal gyrus, inferior frontal gyrus, the medial prefrontal cortex, the entorhinal cortex, the orbitofrontal cortex, the anterior insular cortex, the temporal pole, the primary somatosensory cortex and the inferior parietal cortex in FTD PPA patients as compared to AD PPA patients. AD PPA patients, when compared with the brain metabolism of FTD PPA patients, showed significant hypometabolism in the superior parietal lobule, the inferior parietal lobule, the angular gyrus, the posterior cingulate cortex, the posterior paracentral lobule, the precuneus, the parahippocampal gyrus, the cerebellar vermis and the cerebellar declive, folium, and pyramis. Significant differences in glucose metabolism between subjects suffering from FTD PPA and AD PPA can be observed in Fig. 4.

### NOSE-tool

The here developed and tested diagnostic tool called NOSE (Neurodegeneration of Olfactory Structures in dEmentia) was made publicly available for individual use on https://nose-tool.com/. The NOSE-tool enables other, independent clinicians and researchers to test and validate the here discussed tool in patients suffering from a neurodegenerative disorder which is suspected to reflect either AD or FTD. We encourage readers to use this tool especially to investigate the discriminatory effect of the NOSE-tool in patients with a logopenic PPA pattern who suffered from either FTD or AD. We urge readers to take notion of the disclaimer prior to usage (see https://nose-tool.com/).

## Discussion

This study showed that the use of [^18^F]-FDG PET to assess brain regions that are part of the olfactory circuit, can be a valid additional tool to differentiate bvFTD and AD patients. To distinguish these neurodegenerative disorders, Z-scores of the amygdala, insular cortex and olfactory cortex showed the highest discriminative power, yielding a sensitivity and specificity of 88 and 82%, respectively. This diagnostic accuracy partially overlaps with the diagnostic accuracy of a classical pattern visible on [^18^F]-FDG-PET to distinguish AD from bvFTD. It must be emphasized that in the current study, the canonical regions involved in bvFTD and AD were not necessarily included as this study aimed to investigate differences in metabolism of brain regions involved in olfactory (dys)function. Nestor et al. described a sensitivity and specificity ranging between 80 and 99% and 63–98%, respectively, when differentiating between bvFTD and AD when examining the canonical patterns [45]. In the current study, SPM analysis revealed that the metabolic rate of the entorhinal cortex was only significantly decreased in PPA of FTD origin, explaining the high diagnostic accuracy of the ROI analysis.

One may appreciate that the AUCs for individual regions are rather poor, as illustrated in Fig. 2B, but the diagnostic accuracy significantly improved when a combination of regions was analysed. The most plausible explanation for this phenomenon is the concept of recognizing metabolic patterns in dementia imaging. Dementias affect the brain as a whole rather than targeting focal areas, necessitating an interpretation of brain metabolism in its entirety. Consequently, focal ROIs inherently exhibit poor diagnostic accuracy for this disease type, as they do not reflect the pathophysiological background. When regions are combined, the here developed diagnostic model more accurately captured the global impact of dementia. For this reason, diagnostic [^18^F]FDG-PET imaging traditionally emphasizes distinct metabolic patterns to differentiate between causes of dementia, rather than individual ROIs [18]. This paradigm was further reflected in the diagnostic accuracy of AD versus FTD differentiation in individual canonical regions. The balanced accuracy (calculated as the average of sensitivity and specificity values) and AUC statistics for the frontal cortex, temporal cortex, medial temporal cortex, parietal cortex, occipital cortex, and cingulate island sign ratio were 58%/59%, 55%/58%, 63%/69%, 65%/67%, 58%/58%, and 54%/51%, respectively. However, when these regions were analysed in combination, the balanced accuracy and AUC values increase to 76% and 78%, respectively, demonstrating a substantial increase in diagnostic power when patterns are considered rather than individual ROIs [46].

Tau-related pathology has been found in the olfactory bulbs of patients with AD, Parkinson’s disease, dementia with Lewy bodies, and bvFTD, although this has not been observed in patients suffering from other disorders which are not clinically associated with olfactory dysfunction, such as progressive supranuclear palsy and corticobasal degeneration [6]. Furthermore, in AD patients it has recently been suggested that decreased ability to identify odors may reflect the burden of tau-mediated neurodegeneration in the mesotemporal lobe (i.e., amygdala, hippocampus and parahippocampal gyrus) [47]. It is also known that these regions are among the first to show tau pathology and correspond to early Braak stages [48] – particularly Braak II and later stages [49]- and could therefore help in the early detection of AD and other neurodegenerative disorders. To the authors’ knowledge, no previous investigations showed hypometabolism within the regions of the olfactory circuit as assessed by [^18^F]-FDG PET imaging. In addition, to use these regions to distinguish normal metabolism from abnormal metabolic signatures has not been described. Furthermore, the here described differentiation between bvFTD and AD solely on the use of metabolic patterns of the olfactory circuit could help explain the different clinical phenotypes of olfactory dysfunction described in neurodegenerative patients. In bvFTD, patients seem to present with alterations in odor identification, although odor discrimination seems preserved, indicating that in these patients, the deficits might represent cognitive alterations more than sensory deficits [11]. In AD subjects (Braak V stage or greater), on the other hand, post-mortem investigation of the olfactory bulbs showed deposits of amyloid-β and/or α-synuclein [50]. This neuropathological biomarker, in turn, has been related to synaptic dysfunction of the olfactory system, indicating neuronal damages causing the olfactory deficits [51]. Based on these neuropathological and clinical characteristics, current results can be explained, hypothesizing that olfactory dysfunction in AD patients is explained by a sensory deficit of the olfactory circuit.

### Strengths and limitations

Comparable to other [^18^F]-FDG-PET imaging studies, it is known that SUVR measurements form a limitation of this type of research. Previous research demonstrated that SUVR measurements can be affected by a variety of factors, including quality of injection of the radiotracer, patient cooperation during the uptake time, accuracy of activity measurement and scanner calibration. The homogeneous scanning protocol and the fact that all scanning was performed on the same scanning system largely evades these possible limitations. Another limitation of the present study concerns the limited sample size of each group of patients and the retrospective nature of this study. Regarding the SPM analysis, a limitation was the use of a discrete cosine transform model as inter-subject alignment through the DARTEL algorithm is more accurate than discrete cosine transform models [52, 53]. Albeit, these differences in algorithm performance have been found in MRI data and as this study did not incorporate MRI data, the use of the DARTEL algorithm for spatial normalization was not suitable. A strength concerns the use of the voxel-to-voxel basis comparison of different regions of interest which were based on the Talairach coordinate system. By using this system, the metabolism in near-identical brain regions, which were considered to represent the elements of the olfactory circuit, could be assessed.

The balanced composition of the patient cohort diagnosed with PPA can be considered both a strength and a limitation. On one hand, achieving reasonably even groups enhances the statistical power of t-test comparisons, particularly within the framework of SPM analysis, thus contributing to the robustness of the results. The deviation from the typical epidemiological distribution of PPA does not affect the metabolic signature identified in our findings. However, this deviation may influence the sensitivity and specificity values, as well as the practical application of the developed NOSE tool. Nevertheless, we believe that the impact on the accuracy of the NOSE tool is minimal.

Another relative limitation of this study concerns the missing of clinical characteristics of patients, including that none of the included patients underwent systematic olfactory function testing. However, olfactory deficits associated with neurodegenerative disorders generally remain subclinically [54]. Thereby, the use of olfactory tests with their poor diagnostic accuracy and poor predictive values, is much debated [55–58]. Therefore, the impact of this omission is regarded as very limited as it would not have provided relevant patient information. Similarly, as this study did not focus on cognitive function, including psychometric test results (e.g., mini-mental state examination results) would not have contributed in a relevant way to the dataset used for this study.

## Conclusion

Glucose metabolism of subdivisions of the olfactory circuit as assessed by [^18^F]-FDG PET brain imaging can be used to distinguish AD and bvFTD from normal subjects and from each other. Furthermore, significant different patterns of hypometabolism can be observed between AD PPA patients and FTD PPA patients. More research is needed to assess how these metabolic changes reflect different neuronal mechanisms which drive this clinical symptom of heterogeneous origin.

## Data Availability

No datasets were generated or analysed during the current study.
